# Inhibitory Effect of Nasal Intermittent Positive Pressure Ventilation on Gastroesophageal Reflux

**DOI:** 10.1371/journal.pone.0146742

**Published:** 2016-01-19

**Authors:** Danny Cantin, Djamal Djeddi, Vincent Carrière, Nathalie Samson, Stéphanie Nault, Wan Lu Jia, Jennifer Beck, Jean-Paul Praud

**Affiliations:** 1 Neonatal Respiratory Research Unit, Department of Pediatrics, Université de Sherbrooke, Sherbrooke, Quebec, Canada; 2 Department of Physiology, Université de Sherbrooke, Sherbrooke, Quebec, Canada; 3 Department of Pediatrics, Université Picardie Jules Verne, Amiens, France; 4 Department of Pediatrics, University of Toronto, Toronto, Ontario, Canada; 5 Keenan Research Centre for Biomedical Science of St. Michael’s Hospital, Toronto, Ontario, Canada; 6 Institute for Biomedical Engineering and Science Technology (iBEST) at Ryerson University and St-Michael’s Hospital, Toronto, Ontario, Canada; Hôpital Robert Debré, FRANCE

## Abstract

Non-invasive intermittent positive pressure ventilation can lead to esophageal insufflations and in turn to gastric distension. The fact that the latter induces transient relaxation of the lower esophageal sphincter implies that it may increase gastroesophageal refluxes. We previously reported that nasal Pressure Support Ventilation (nPSV), contrary to nasal Neurally-Adjusted Ventilatory Assist (nNAVA), triggers active inspiratory laryngeal closure. This suggests that esophageal insufflations are more frequent in nPSV than in nNAVA. The objectives of the present study were to test the hypotheses that: i) gastroesophageal refluxes are increased during nPSV compared to both control condition and nNAVA; ii) esophageal insufflations occur more frequently during nPSV than nNAVA. Polysomnographic recordings and esophageal multichannel intraluminal impedance pHmetry were performed in nine chronically instrumented newborn lambs to study gastroesophageal refluxes, esophageal insufflations, states of alertness, laryngeal closure and respiration. Recordings were repeated without sedation in control condition, nPSV (15/4 cmH_2_O) and nNAVA (~ 15/4 cmH_2_O). The number of gastroesophageal refluxes recorded over six hours, expressed as median (interquartile range), decreased during both nPSV (1 (0, 3)) and nNAVA [1 (0, 3)] compared to control condition (5 (3, 10)), (p < 0.05). Meanwhile, the esophageal insufflation index did not differ between nPSV (40 (11, 61) h^-1^) and nNAVA (10 (9, 56) h^-1^) (p = 0.8). In conclusion, nPSV and nNAVA similarly inhibit gastroesophageal refluxes in healthy newborn lambs at pressures that do not lead to gastric distension. In addition, the occurrence of esophageal insufflations is not significantly different between nPSV and nNAVA. The strong inhibitory effect of nIPPV on gastroesophageal refluxes appears identical to that reported with nasal continuous positive airway pressure.

## Introduction

Non-invasive ventilatory support, either continuous (nCPAP) or intermittent (nIPPV) positive airway pressure, has been associated with the insufflation of gas into the esophagus and in turn to gastric distension in subjects of all ages [1–4]. Moreover, air-induced gastric distension is considered to be a major mechanism responsible for transient relaxation of the lower esophageal sphincter [5]. Non-invasive ventilatory support, when responsible for gastric distension, may hence be responsible for increasing gastroesophageal refluxes (GER) [4]. Yet, the consequences of non-invasive ventilatory support on GERs appear more complex. Indeed, we have previously shown that application of an nCPAP of 6 cmH_2_O, which did not appear to be associated with gastric distension, virtually abolished GERs in lambs [6]; importantly, this result is in agreement with previous reports on nCPAP in adult humans [7]. However, given that peak inspiratory pressure during nIPPV is most often well above 6 cmH_2_O, even in newborns [3], it would thus seem logical to assume that nIPPV will further promote gastric distension and GERs compared to nCPAP.

Moreover, we have previously shown that certain nIPPV modes, such as Pressure Support Ventilation (nPSV), lead to active glottal closure against ventilator insufflations in about 75% of lambs at and above an inspiratory pressure of 15 cmH_2_O [8,9]. A similar observation has also been previously made *via* endoscopy in adult humans [10]. These consistent observations led us to assume that active glottal closure in nPSV will promote esophageal insufflations and gastric distension, thereby further increasing GERs. In contrast, our observation that another nIPPV mode, namely nasal Neurally-Adjusted Ventilatory Assist (nNAVA), did not lead to active glottal closure [11] led us to hypothesize that nNAVA would not increase (or to a lesser degree) GERs.

To the best of our knowledge, there are no current data specifically addressing the effect of nIPPV on GERs and no direct documentation of esophageal insufflations during nIPPV. Thus, the present study first aimed to test the two-fold hypothesis that i) compared to control condition (= no nIPPV), GERs are increased during nIPPV performed at a peak inspiratory pressure (15 cmH_2_O) commonly used, in the neonatal period [3] and beyond and ii) the increase in GERs is higher in nPSV compared to nNAVA. In addition, we aimed to provide direct documentation of esophageal insufflations during nIPPV and to investigate whether: i) esophageal insufflations are increased in nPSV compared to nNAVA; ii) the number of esophageal insufflations is related to the number of GERs in nPSV and nNAVA, and iii) esophageal insufflations during nPSV are increased during the period of active laryngeal closure.

Our results highlight that both nPSV and nNAVA significantly inhibit GERs at the pressures used in the present study, and that esophageal insufflations are not more prominent in nPSV vs. nNAVA or when laryngeal closure is present.

## Materials and Methods

Nine mixed-bred full-term lambs, born by spontaneous vaginal delivery, aged 4 to 5 days and weighing 4.6 ± 0.9 kg (mean ± SD) were included in the study. Based on our extensive experience of over 25 years in such experiments, this number was deemed appropriate to provide sufficient statistical power and represent a good compromise between the need to include a sufficient number of lambs to answer our main research question and the need to reduce the number to a minimum to meet ethical requirements. The study was approved by the Ethics Committee for Animal Care and Experimentation of the Université de Sherbrooke (protocol # 283–11).

### Chronic Instrumentation and Experimental Equipment

Chronic surgical instrumentation was performed under general anesthesia (2% isoflurane) and included insertion of i) custom-built bipolar electrodes into both thyroarytenoid (ta, a laryngeal constrictor) muscles for recording of electrical activity (EAta) and ii) a catheter into the left carotid artery for blood gas analysis [11]. Before surgical instrumentation, one dose of ketoprofen (3 mg/kg intramuscularly) was administered for analgesia; the same dose was repeated if needed on the day following surgery.

Lamb instrumentation was completed immediately before recordings. Needle electrodes were inserted subcutaneously for electroencephalogram (EEG) and electrooculogram (EOG) recordings. Elastic bands were installed on the chest and abdomen to monitor lung volume variations semi-quantitatively *via* respiratory inductance plethysmography. Continuous monitoring of oxygen hemoglobin saturation (SpO_2_) was performed using a Masimo pulse oximeter placed at the base of the tail.

Two different modes of synchronized nIPPV, namely nPSV and nNAVA, were administered using a Servo-i ventilator (Maquet Critical Care, Solna, Sweden) through a custom-built nasal mask filled with dental paste to fit the lamb’s muzzle and prevent unintentional leaks [12]. The nasal mask was not installed for the control condition (= spontaneous breathing without nIPPV). An 8-Fr NAVA catheter was nasally inserted into the esophagus down to the level of the crural diaphragm as previously described [13]. This catheter contains an array of miniaturized sensors for measurement of the crural diaphragm electromyogram (EAdi). During nNAVA, the latter was captured and fed to the Servo-i ventilator in order to deliver a ventilatory assistance in proportion to and in synchrony with the lamb’s inspiratory efforts. The level of ventilatory pressure delivered was continuously monitored at the mask (Pmask) and set at 15/4 cmH_2_O for nPSV (= pressure support level of 11 cmH_2_O above a PEEP of 4 cmH_2_O) and equivalently for nNAVA (NAVA gain adjusted between 0.4 and 1.4). Finally, both GERs and esophageal insufflations were continuously assessed *via* a MII-pH catheter (diameter = 2 mm, Unisensor, Portsmouth, USA) inserted nasally, its position being confirmed by an X-ray and secured with sutures [6].

Physiological signals were transmitted wirelessly and continuously recorded on a PC [14]. The entire recording period was also filmed with a webcam, while an experimenter was present to note all events occurring during the recordings. At the end of experiments, lambs were euthanized by pentobarbital overdose (90 mg/kg intravenously).

### Design of the Study

Chronic instrumentation was performed on the day of lamb’s arrival in our animal quarters. After 36h of postoperative recovery, six-hour polysomnographic recordings were performed during nPSV, nNAVA and without nIPPV on three successive days in randomized order. Lambs were placed in a sling throughout the six-hour nIPPV sessions, then immediately returned into a Plexiglas chamber after each nIPPV session, where they were free to move and feed for a further six-hour recording without any ventilatory support. This additional recording aimed to assess whether carryover effects of nIPPV on GERs were present, as previously observed following application of a nCPAP of 6 cmH_2_O [6].

### Data Analysis

#### States of alertness

Standard electrophysiological and behavioral criteria were used to define quiet (QW) and active (AW) wakefulness, as well as quiet sleep (QS) and active sleep (AS) [15].

#### Gastroesophageal refluxes

MII-pH recordings were automatically analyzed with the MMS software (Medical Measurement Systems USA Inc, Dover, NH) and visually verified. The number of GERs per hour of total recording and per hour of each state of alertness, as well as the total number of GERs during the six-hour recording periods, was calculated. Characterization of GERs as liquid, purely gaseous or mixed, as well as their pH, was performed according to published criteria [16]. In addition, the bolus exposure index (BEI; % total time spent in liquid reflux), the bolus clearance time (time required for the entire bolus to exit the esophagus) and the number of proximal GERs (= GERs reaching the most rostral channel of the MII-pH) were calculated.

#### Esophageal insufflations during nIPPV

(See S1 Text). *Frequency of esophageal insufflations*: MII-pH recordings were also used to count the number of esophageal insufflations and to calculate the esophageal insufflation index (= number of esophageal insufflations per hour), as well as the percentage of respiratory cycles with esophageal insufflation for the entire recording period in each of the three respiratory conditions. The effect of the state of alertness on esophageal insufflations was also assessed.

*Effects of esophageal insufflations on GERs*: A correlation analysis was performed to test whether the number of esophageal insufflations was associated with the total number of GERs or the number of gas-containing (gaseous + mixed) GERs observed during the six-hour recording with nPSV and nNAVA.

*Effect of active laryngeal closure on esophageal insufflations*: Lastly, the potential effect of inspiratory EAta, i.e., active glottal closure against ventilator insufflations, on diverting the insufflated gas into the esophagus during nPSV was assessed. Following confirmation that EAta was less frequent during AS, the % of respiratory cycles with esophageal insufflations was compared between QS and AS. Such an assessment could not be performed in nNAVA, due to the absence of inspiratory EAta as previously reported [11].

### Statistical Analysis

Normality was systematically tested using the Shapiro-Wilk test. All data were not normally distributed and hence expressed as median and interquartile range (Q1, Q3). Statistical analyses were performed on raw data for all dependent variables. Comparisons between different conditions were carried out using either the Wilcoxon signed-rank test or the Friedman test completed by the Wilcoxon signed-rank test as appropriate. Spearman rank correlation was used to test the association between the number of esophageal insufflations and the number of GERs. A p value < 0.05 was chosen to determine statistical significance. The statistical analyses were performed using SPSS software (version 22, Chicago, IL, USA).

## Results

### Recordings during Non-Invasive Ventilation

Measurements of peak inspiratory pressures during one minute every hour yielded values of 16 (15, 17) cmH_2_O (median (Q1, Q3)) during nPSV and 13 (12, 14) cmH_2_O during nNAVA.

In each state of alertness, there were no significant differences between the percentages of time spent in the three respiratory conditions (S1 Fig).

#### Gastroesophageal refluxes

**Fig 1:**
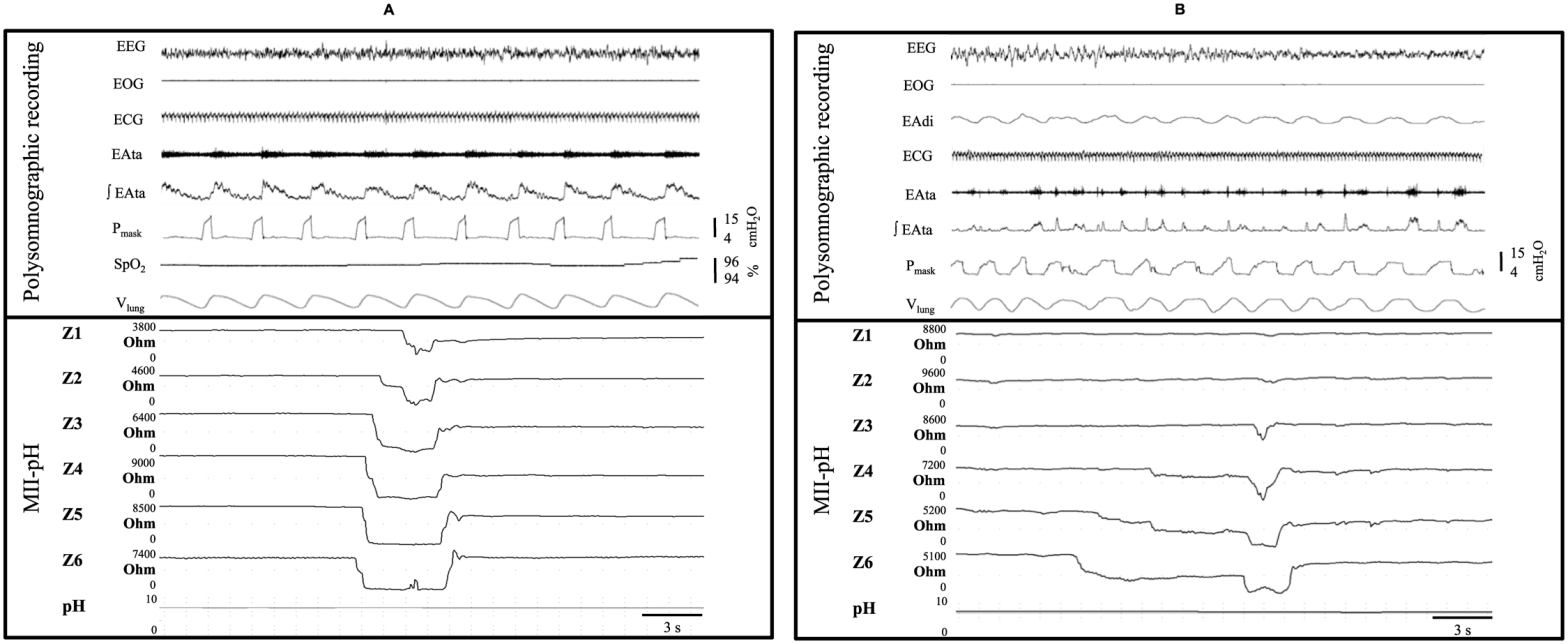
Liquid gastroesophageal reflux during (A) nasal pressure support ventilation (B) nasal neurally-adjusted ventilatory assist. From top to bottom: EEG, electroencephalogram; EOG, electrooculogram; ECG, electrocardiogram; EAdi, electrical activity of the diaphragm; EAta, thyroarytenoid muscle (laryngeal constrictor) electrical activity; ∫EAta, moving time averaged EAta; P_mask_, mask pressure; SpO_2_, pulse oximetry; V_lung_, lung volume variations = sum signal of respiratory inductance plethysmography; Z1 to Z6, impedance channels of the esophageal multichannel intraluminal impedance-pHmetry (MII-pH). The decrease in esophageal impedance progressing from Z6 up to Z1 (the most proximal channel of the MII-pH) illustrates a proximal gastroesophageal reflux.

**Fig 2:**
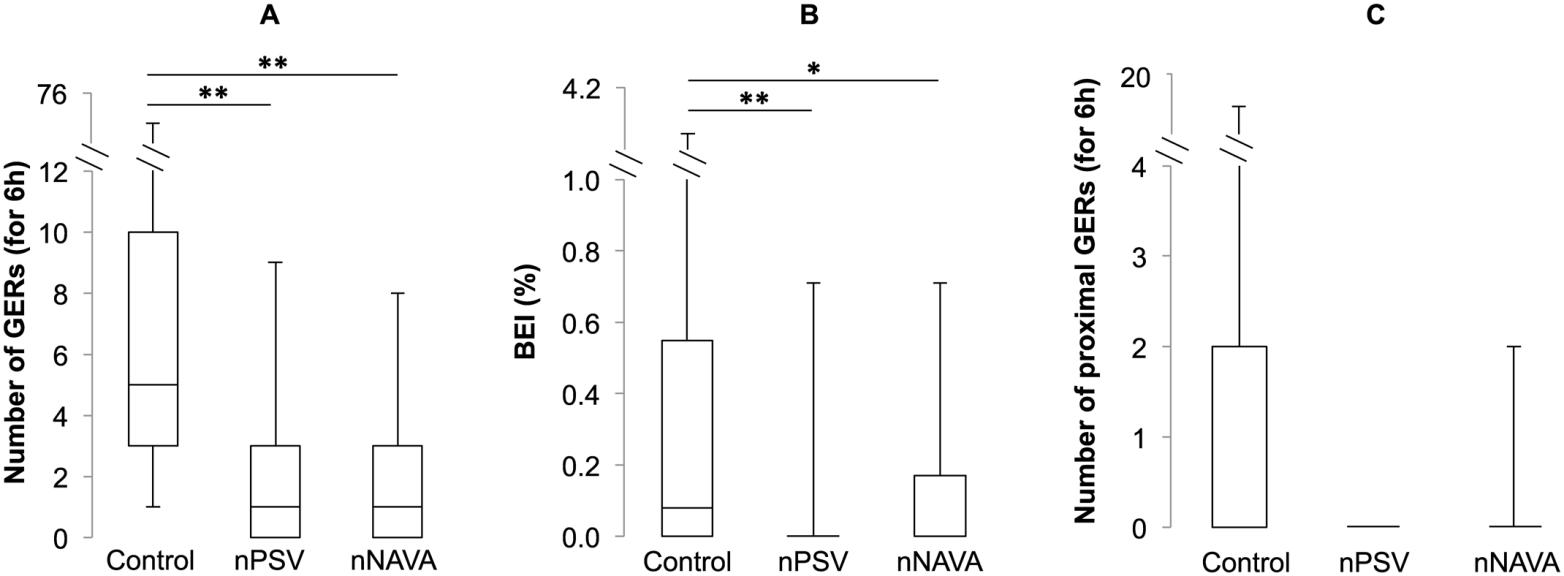
Effects of nasal ventilation on gastroesophageal refluxes. Fig 2-A represents the total number of GERs, 2-B the bolus exposure index and Fig 2-C the number of proximal GERs. Both liquid- and gas-containing gastroesophageal refluxes are included. Box and whisker plots represent interquartile ranges (upper = Q3, middle line = median, bottom = Q1) with maximum and minimum values, respectively. **: p < 0.05; *: p < 0.1.

Fig 1 illustrates the GERs observed during nPSV and nNAVA. Irrespective of the condition, most GERs (77%) were weakly acidic while no acid GERs were observed (S1 Table). Overall, GERs were similarly inhibited during both nPSV and nNAVA (Fig 2A) (nPSV vs. control, p = 0.01; nNAVA vs. control, p = 0.03; nPSV vs. nNAVA, p = 0.7). In addition, contrary to the control condition, no GERs were observed after the first two hours of nPSV or nNAVA (S2 Fig). Moreover, while the frequency of GERs appeared to be lower during both nPSV and nNAVA in all states of alertness, this decrease only reached statistical significance during QW (p < 0.03 for both nPSV and nNAVA vs. control) (S3 Fig).

Similarly to the number of GERs, the overall bolus exposure index decreased in both nPSV (p = 0.02) and nNAVA (p = 0.06) compared to the control condition (Fig 2B). Meanwhile, bolus clearance time did not differ significantly between the three conditions (p = 0.1). Finally, no significant differences were observed between the three conditions with regard to proximal GERs (p = 0.7) (Fig 2C).

#### Esophageal insufflations during nIPPV

**Fig 3:**
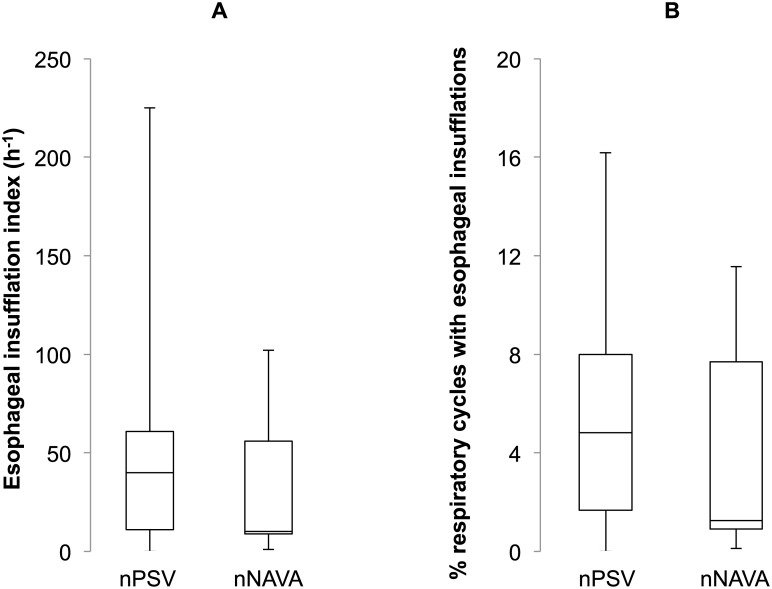
Frequency of esophageal insufflations during nasal pressure support ventilation and nasal neurally-adjusted ventilatory assist. The esophageal insufflation index (number of insufflations per hour) (A), as well as the % of respiratory cycles with esophageal insufflations (B), was not significantly different in nasal pressure support ventilation and nasal neurally adjusted ventilatory assist.

(See S2 Text). *Frequency of esophageal insufflations*: No gas swallows were observed in control condition in any of the lambs. Conversely, esophageal insufflations were observed in both nPSV and nNAVA in most lambs, with no significant differences overall in the esophageal insufflation index between nPSV (40 (11, 61) h^-1^) and nNAVA (10 (9, 56) h^-1^) (p = 0.8) (Fig 3A). This corresponded to a low percentage of respiratory cycles with esophageal insufflation, which again was not significantly different between nPSV (5 (2, 8)%) and nNAVA (1 (1, 8)%) (p = 0.9) (Fig 3B).

**Fig 4:**
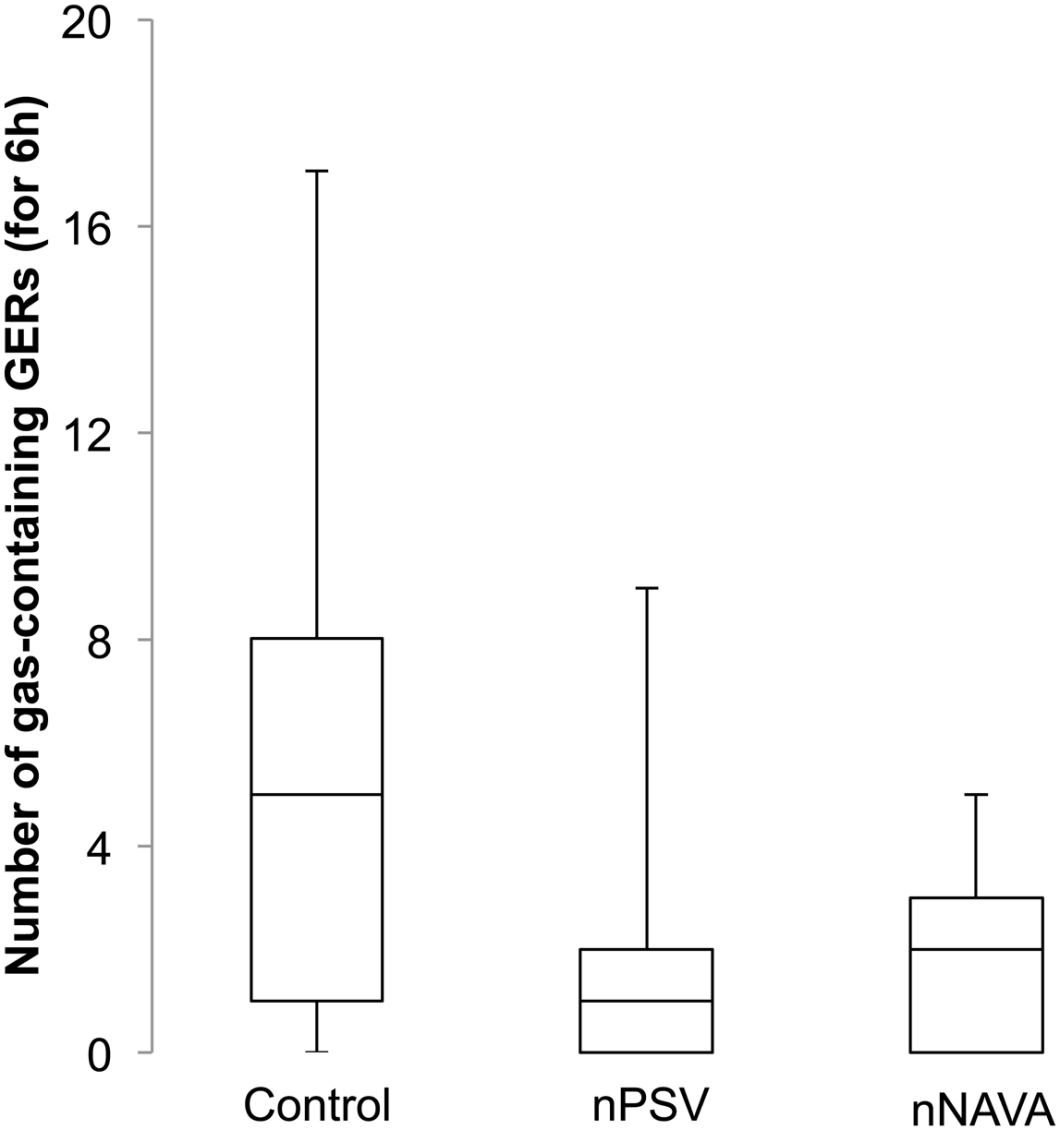
Number gas-containing gastroesophageal refluxes with nasal pressure support ventilation and nasal neurally-adjusted ventilatory assist. Despite the presence of esophageal insufflations during nasal pressure support ventilation (nPSV) and nasal neurally adjusted ventilatory assist (nNAVA), but not during the control condition, no significant increase in gas-containing gastroesophageal refluxes was observed in nPSV or nNAVA compared to the control condition.

*Effects of esophageal insufflations on GERs*: Despite the insufflation of air into the esophagus during nPSV and nNAVA, no increase was observed in nPSV or nNAVA with regard to the number of gas-containing (gaseous + mixed) GERs compared to the control condition (p = 0.1) (Fig 4). In addition, no significant association was found between the number of total GERs, or the number of gas-containing GERs, and the number of esophageal insufflations (S4 Fig).

Furthermore, the absence of increase in abdominal circumference during nIPPV sessions suggested that neither nPSV nor nNAVA was responsible for gastric distension.

**Fig 5:**
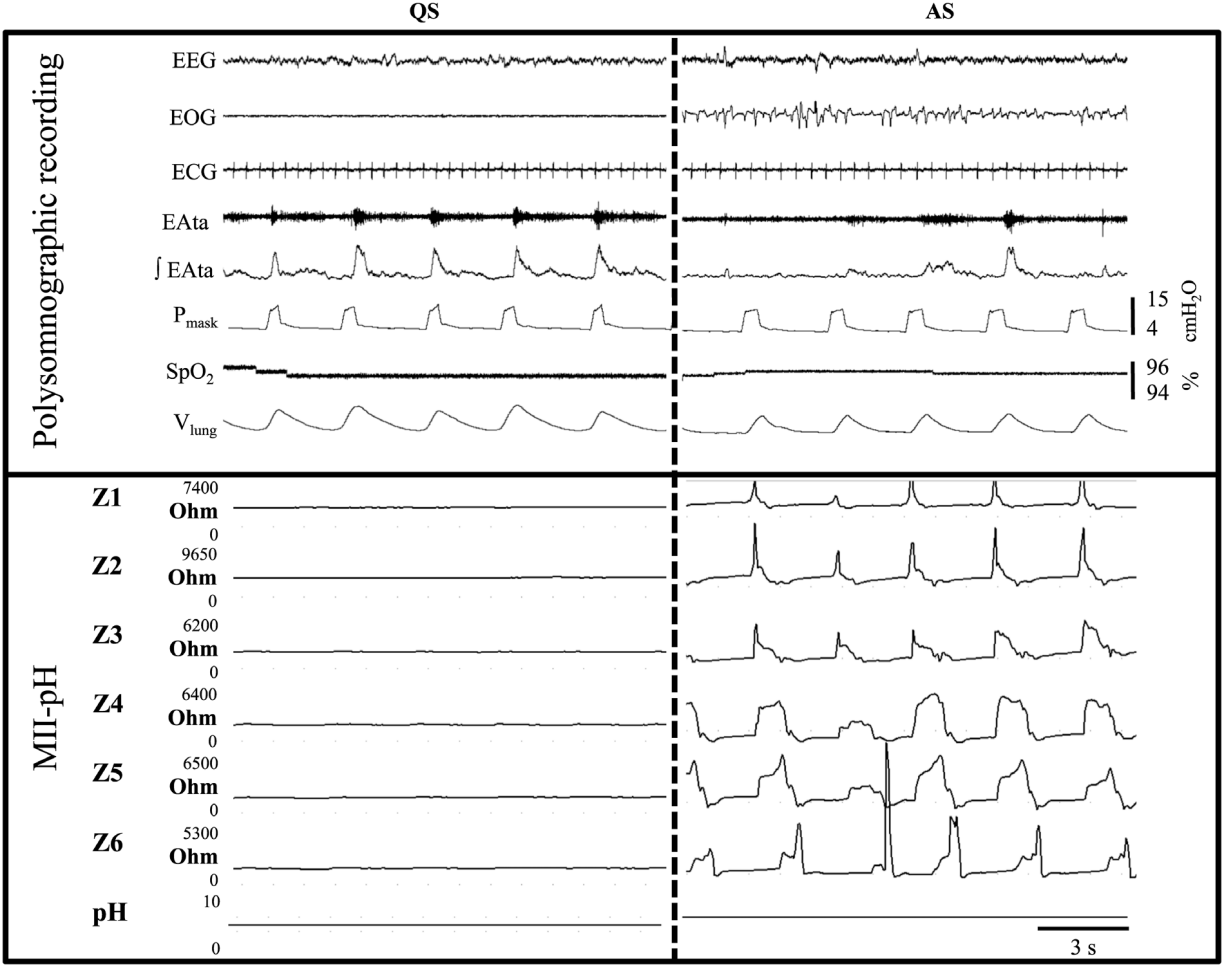
Esophageal insufflations during nasal pressure support ventilation in quiet and active sleep. Two successive periods of quiet and active sleep in the same lamb are represented. The most frequent pattern of esophageal insufflation and EAta recording is illustrated. In active sleep (AS), all five successive ventilator insufflations (see mask pressure) were accompanied by an increase in esophageal impedance progressing from the oral (Z1) down to caudal (Z6) channels of the MII-pH, depicting the insufflation of gas into the esophagus (= esophageal insufflations); in this example, esophageal insufflations were present in AS only. See Fig 1 for abbreviations.

**Fig 6:**
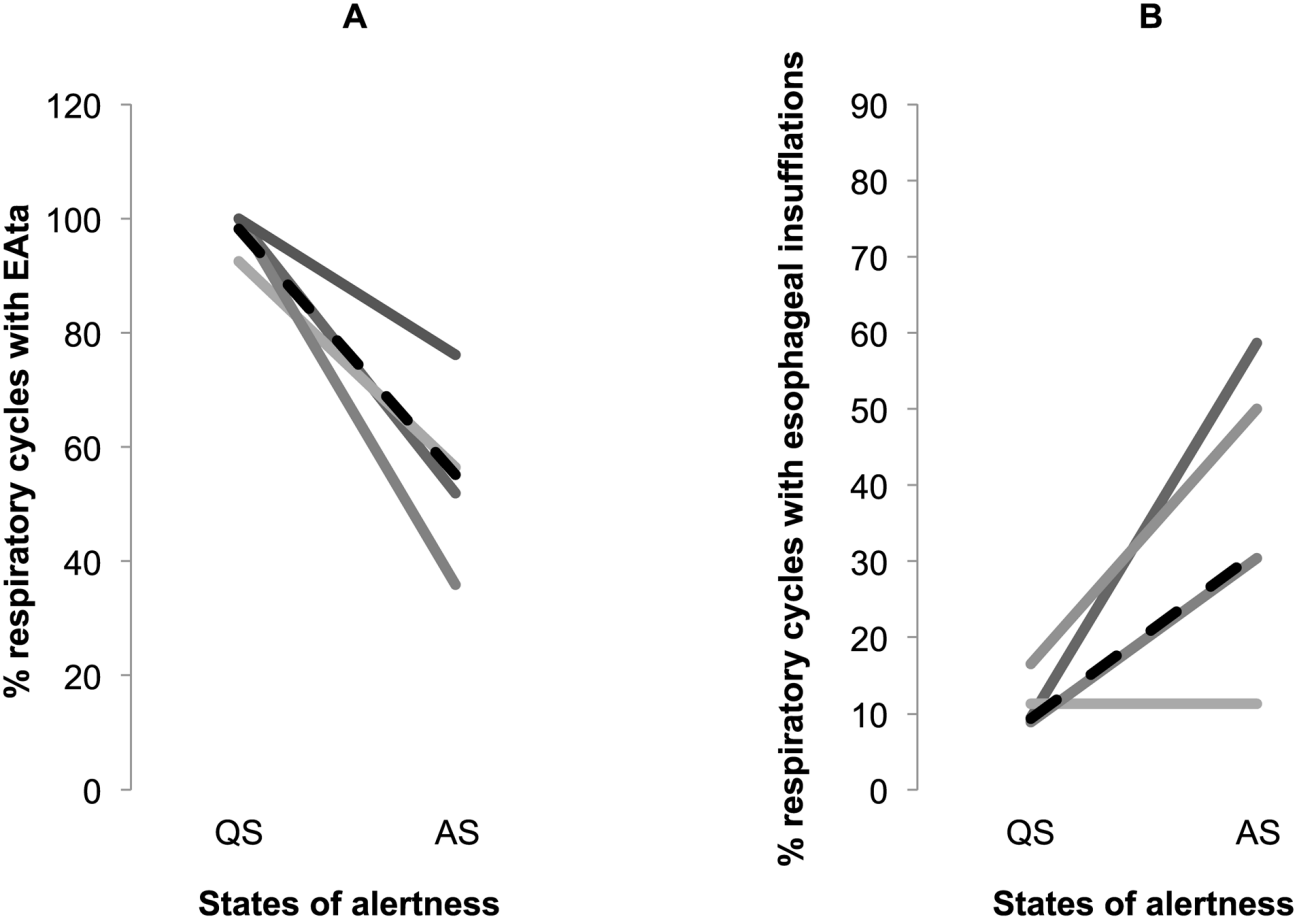
Effects of active sleep on active inspiratory laryngeal closure and esophageal insufflations. The effect of active sleep is illustrated only during nasal pressure support ventilation, for inspiratory laryngeal closure is not observed in nasal neurally-adjusted ventilatory assist. While the transition from quiet sleep (QS) to active sleep (AS) decreased the % of respiratory cycles with active laryngeal closure (p = 0.07, n = 4) (A), it conversely increased the % of respiratory cycles with esophageal insufflations (p = 0.07, n = 4) (B), strongly suggesting that laryngeal closure against ventilator insufflations is not the main factor responsible for esophageal insufflations, at least in AS.

*Effect of active laryngeal closure on esophageal insufflations*: The most frequent pattern of esophageal insufflations and EAta recordings obtained during two successive periods of QS and AS is illustrated in Fig 5. The percentage of respiratory cycles with inspiratory EAta (an evidence of active laryngeal closure) in nPSV was first confirmed to be lower in AS compared to QS (54 (48, 61)% vs. 100 (98, 100)% respectively, p = 0.07; n = 4) (Fig 6A). In contrast, the % of respiratory cycles with esophageal insufflation was higher in AS (30 (11, 50)% compared to QS (4 (1, 15)%, p = 0.07; n = 4) (Fig 6B), suggesting that esophageal insufflations were not the result of active laryngeal closure.

### Recordings during the Six-Hour Post-nIPPV Period

The vast majority of GERs (93%) were again weakly acidic with no acid GERs observed during the post-nIPPV period. Conversely to the six-hour nIPPV period, no significant differences were observed in either the total number of GERs, the bolus exposure index, the number of proximal GERs or the bolus clearance time between the three conditions (p between 0.1 and 0.8). In addition, conversely to observations during the six-hour nIPPV period, results were consistent throughout the post-ventilation period.

## Discussion

To our knowledge, this is the first study to specifically demonstrate the effect of non-invasive positive pressure ventilation on gastroesophageal reflux. Our results indicate that nasal intermittent positive pressure ventilation at 15/4 cmH_2_0 markedly reduced the number of gastroesophageal refluxes in lambs, despite some insufflation of gas into the esophagus. Contrary to our hypothesis, similar results were observed between nasal pressure support ventilation and neurally-adjusted ventilatory assist. This new knowledge opens valuable physiological perspectives in addition to being likely clinically relevant, especially for infants, given both the high prevalence of GERs [17] and the increasing use of non-invasive ventilation in early life [18].

### The Newborn Lamb Model for Studying nIPPV and GERs

Over the past ten years, we have been able to gain unique and extensive expertise in non-invasive ventilatory support in lambs [8,9,11,19,20]. Our team has also been pioneer in GER recordings in a newborn animal model in which GERs can be readily characterized using MII-pH in non-sedated conditions [21]. The preruminant, monogastric newborn lamb has well-established upper and lower esophageal sphincters ([6] and unpublished observations) and presents spontaneous GERs, which bear several features similar to those of the human infant [21]. The use of such an animal model offers a unique means to perform prospective studies where nIPPV conditions can be strictly controlled and randomized in the same subject, thus paving the way for clinical studies.

### Decrease in GERs during nIPPV

There is no direct evidence in the adult or pediatric literature, to our knowledge, of a causal link between IPPV and GERs, whether endotracheal or non-invasive ventilation. The only study which prospectively attempted to assess the effect of non-invasive ventilatory support was performed in premature newborns, albeit with a number of issues which preclude any meaningful conclusion, of which include the assessment of a heterogeneous group of preterms on nIPPV or nCPAP without any distinction, and absence of information on nIPPV mode or pressure level [22].

Several studies have nevertheless reported an inhibition of acid GERs by nCPAP in adult humans [23–25]. Using MII-pH, our team similarly showed a virtual abolition of GERs during nCPAP at 6 cmH_2_O in newborn lambs [6]. Results from the present study extend these previous data on nCPAP by showing that nIPPV also inhibits GERs in lambs, even when the inspiratory pressure is set as high as 15 cmH_2_O.

The mechanisms responsible for the inhibition of GERs by nIPPV remain to be elucidated. In adult humans with nCPAP, a reduction in the duration and depth of TLESRs (transient lower esophageal sphincter relaxation) has been proposed as a key factor [7]. We similarly observed a decreased relaxation of the lower esophageal sphincter during primary peristalsis in lambs on nCPAP [6]. Our current hypothesis is that the same mechanism is at play during nIPPV, insomuch as the inspiratory positive pressure is not sufficiently high to distend the stomach *via* esophageal insufflation. Otherwise, gastric distension would ensue and likely trigger TLESRs, thus increasing GERs [26]. Finally, given that longitudinal esophageal muscle contractions are responsible for initiating TLESRs [27], the caudal displacement of the diaphragm and mediastinum driven by nIPPV may cause an increased length of the esophagus, which would decrease the capacity of the esophageal longitudinal muscle to promote TLESR [7]. Complementary studies are thus clearly needed to unravel the mechanisms underlying this inhibition.

The novel observation that nIPPV decreases weakly acidic GERs is especially relevant to the neonatal period. Indeed, weakly acidic GERs are more prevalent in human infants than acid GERs [16] and are frequently involved in GER disease in early life [28,29].

The observed timeline of GER abolition with non-invasive ventilation is noteworthy. While the number of GERs per hour remained steady during the six-hour control period, the few GERs observed during nPSV and nNAVA occurred only during the first two hours of recording. This observation suggests that the inhibitory effect of nPSV and nNAVA requires a certain amount of time before taking full effect; moreover, when established, this inhibition lasts for several hours.

### Esophageal Insufflations

While it is well known that nIPPV can cause insufflation of gas into the stomach [1,3,4,30], the present study is the first to record and illustrate esophageal insufflations during nIPPV. Overall, the low percentage of respiratory cycles with esophageal insufflations suggests that tonic activity of the upper esophageal sphincter was able to efficiently oppose these insufflations during nIPPV. Esophageal insufflations can however occur *via* pharyngeal reflexive swallowing, which opens the sphincter in response to air injection into the pharynx [31]. Among other factors which may have facilitated esophageal insufflations in our experimental conditions, neither the much shorter inspiratory pressure rise time in nPSV compared to nNAVA [9] nor the higher occurrence of active inspiratory laryngeal closure in QS compared to AS appeared to be involved in this process. Continuous recording of both the tonic and inspiratory electrical activity of the cricopharyngeus muscle (= upper esophageal sphincter), together with pharyngeal and esophageal pressures, may ultimately provide further insight into the determinants of esophageal insufflations during various modes and pressure levels of nIPPV.

Despite the presence of esophageal insufflations during nPSV and nNAVA, the number of gas-containing GERs were not increased in nPSV and nNAVA compared to the no-nIPPV control condition. If anything, the latter was associated with a higher number of gas-containing GERs. Explanation of this observation may again be related to the inhibition of TLESR by nIPPV. Furthermore, the absence of any increase in abdominal perimeter suggests that the total volume of air insufflated into the esophagus was not extensive and air did not accumulate in the stomach, but rather traveled further along the digestive tract. The present findings, together with unpublished personal observations in lambs showing that gastric distension induced by direct insufflations of air into the stomach readily increases gas-containing GERs, lead us to propose that overall, all types of GERs are inhibited by nIPPV, as long as the latter does not induce gastric distension.

### Post-nIPPV Period

Unlike the previously-observed residual effect of nCPAP on GER inhibition [6], the results of the present study suggest that the inhibition of GERs by nPSV and nNAVA does not persist after the withdrawal of ventilation. We have no current explanation for this apparent difference between the effect of nCPAP and nasal ventilation.

### Study Limitations

Certain limitations of the present study should be recognized. First, despite our attempt to match the inspiratory pressure in nNAVA and nPSV, the average inspiratory pressure in nNAVA was slightly lower than in nPSV. This is not surprising since prevention of a high inspiratory pressure is inherent to the nNAVA mode itself. Nevertheless, given that similar effects of nPSV and nNAVA were observed on both GERs and the percentage of respiratory cycles with esophageal insufflations, we do not believe that this difference in inspiratory pressure significantly impacts the present results. Secondly, the design of the mask used in the present study does not allow air leaks during nIPPV; it is unknown whether the effects on GERs would be identical in the presence of significant air leaks. Thirdly, given the paucity of AS periods which can be recorded in lambs during the day, statistical analyses in AS lacked power and could only yield results with a tendency towards statistical significance. Thus, while these latter results are noteworthy, they must nonetheless be considered as preliminary.

From a clinical standpoint, the study was conducted in healthy lambs and for a period of 6 hours only. This is at variance with patients requiring nIPPV for a respiratory compromise lasting several days or more. In addition, the present results are only valid for a pressure level of 15/4 cmH_2_O, which appears insufficient to distend the stomach via esophageal insufflation in healthy newborn lambs. We have previously observed gastric distension in some lambs at higher pressures (20/4 cmH_2_O), which would likely induce TLESR and increase GERs [32].

## Conclusions

Results from the present study and previous knowledge strongly suggest that both nasal CPAP and nIPPV can have a strong inhibitory effect on GERs insofar as they do not induce gastric distension. Pending similar observations in humans, we propose that the present observations may be of significant clinical importance in patients under nIPPV.

## Supporting Information

S1 Fig Percentage of time spent in each respiratory condition and in each state of alertness during the six-hour ventilation period. AS, active sleep; QS, quiet sleep; QW, quiet wakefulness; AW, active wakefulness. See Fig 4 for other abbreviations.
(PDF)

S2 Fig Variation over time of the number of gastroesophageal refluxes (GERs) per hour during the six-hour non-invasive ventilation period. See Fig 4 for abbreviations.
(PDF)

S3 Fig Effect of nasal Pressure Support Support Ventilation (nPSV) and nasal Neurally-Adjusted Ventilatory Assist (nNAVA) on the number of gastroesophageal refluxes (GERs) per hour in each state of alertness during the six-hour ventilation period. See S1 Fig for abbreviations.
(PDF)

S4 Fig Absence of relationship between the number of esophageal insufflations and the number of gastro-esophageal refluxes (GERs) during nasal Pressure Support Ventilation (nPSV) and nasal Neurally-Adjusted Ventilatory Assist (nNAVA). (PDF)

S1 Table Total number and physicochemical characteristics of gastro-esophageal refluxes (GERs) for each lamb during the six-hour ventilation period under control, nasal Pressure Support Ventilation and nasal Neurally-Adjusted Ventilatory Assist conditions. (DOCX)

S1 Text S1 Text contains further detailed description of the analysis that was carried out for esophageal insufflations during nIPPV. (DOCX)

S2 Text S2 Text gives further additional details on the results obtained for esophageal insufflations during nIPPV. (DOCX)
