# Supplementary figures and images for: Inhibitory Effect of Nasal Intermittent Positive Pressure Ventilation on Gastroesophageal Reflux
Source: PLoS One. 2016 Jan 19;11(1):e0146742. doi: 10.1371/journal.pone.0146742 (PMC4718652; doi:10.1371/journal.pone.0146742)

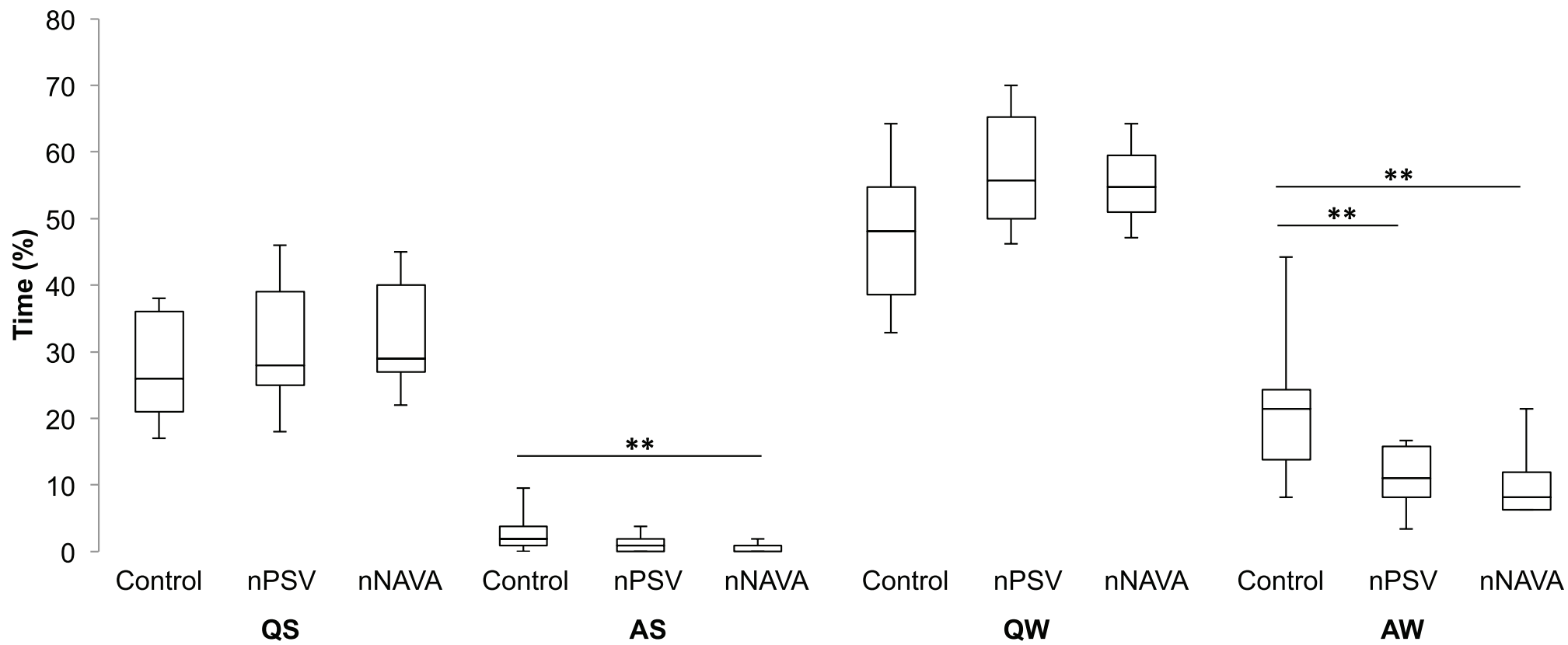

Supplement: S1 Fig — AS, active sleep; QS, quiet sleep; QW, quiet wakefulness; AW, active wakefulness. See Fig 4 for other abbreviations. (PDF) [file pone.0146742.s001.pdf]

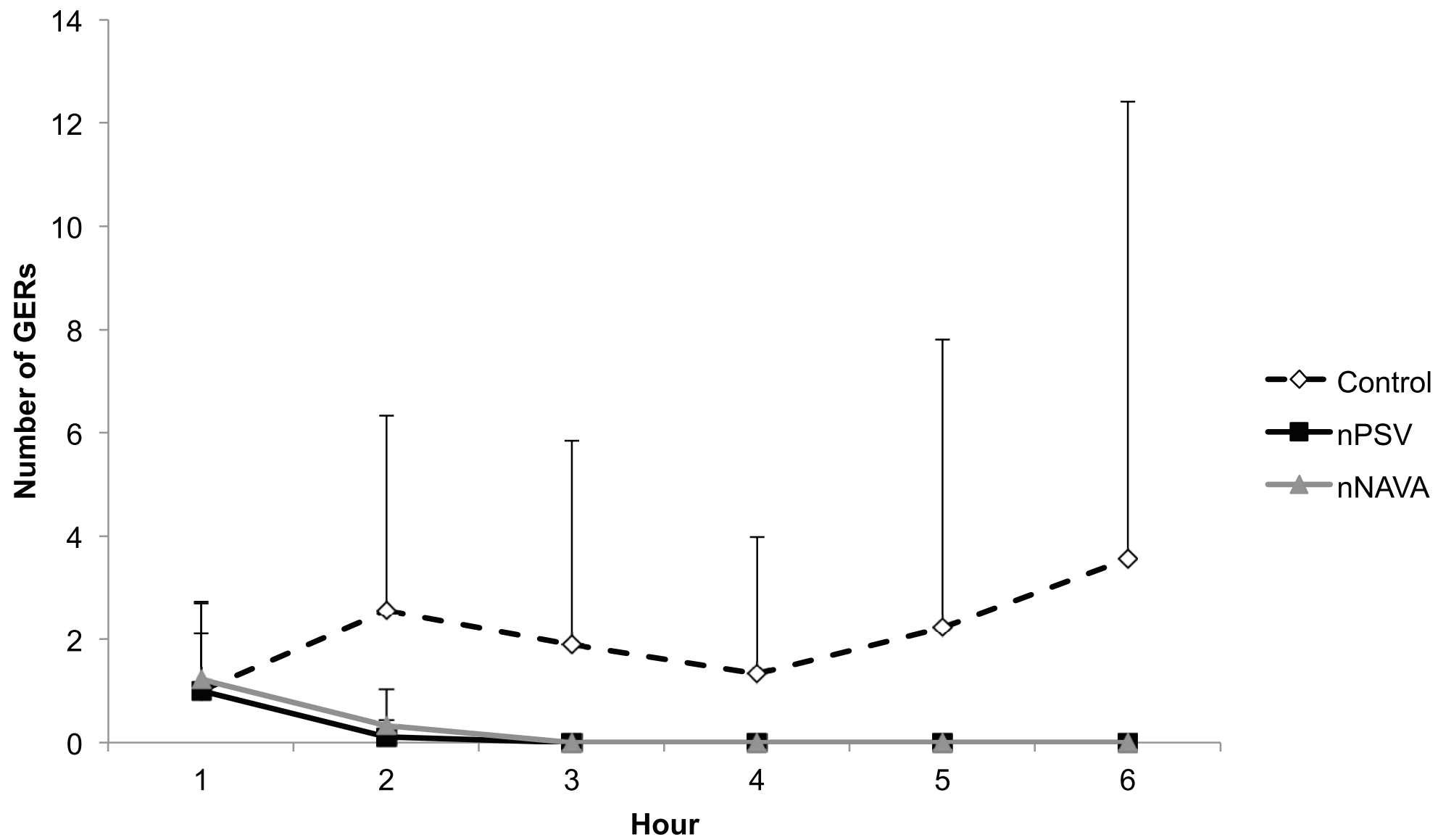

Supplement: S2 Fig — See Fig 4 for abbreviations. (PDF) [file pone.0146742.s002.pdf]

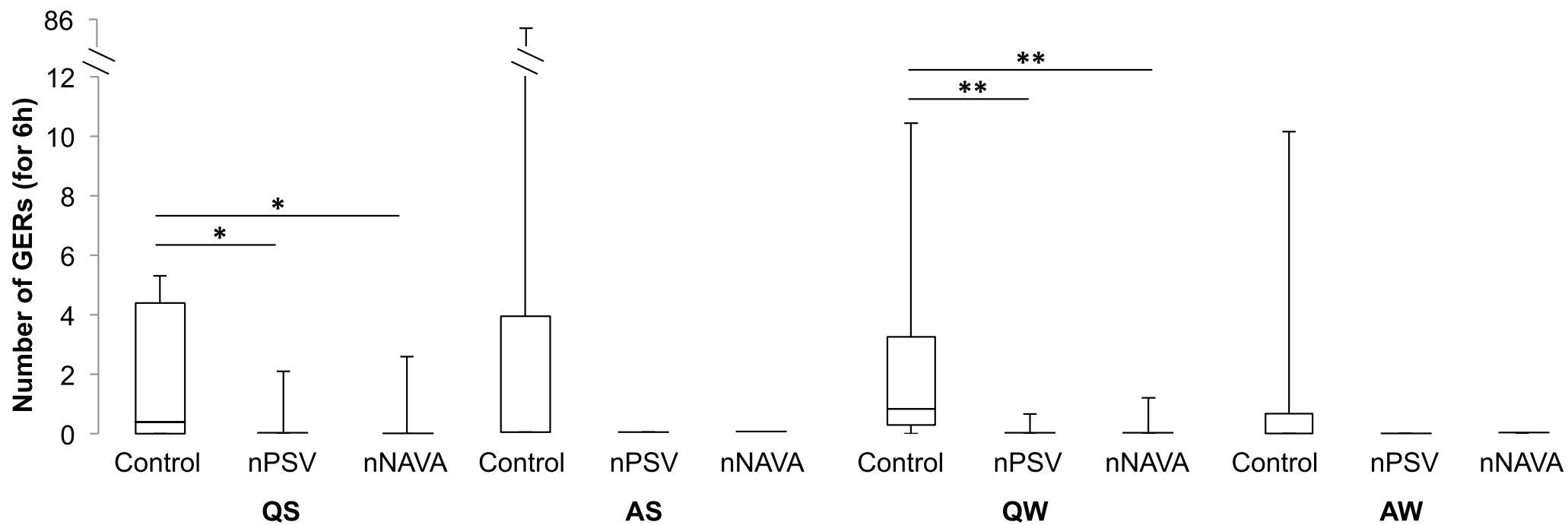

Supplement: S3 Fig — See S1 Fig for abbreviations. (PDF) [file pone.0146742.s003.pdf]

**A**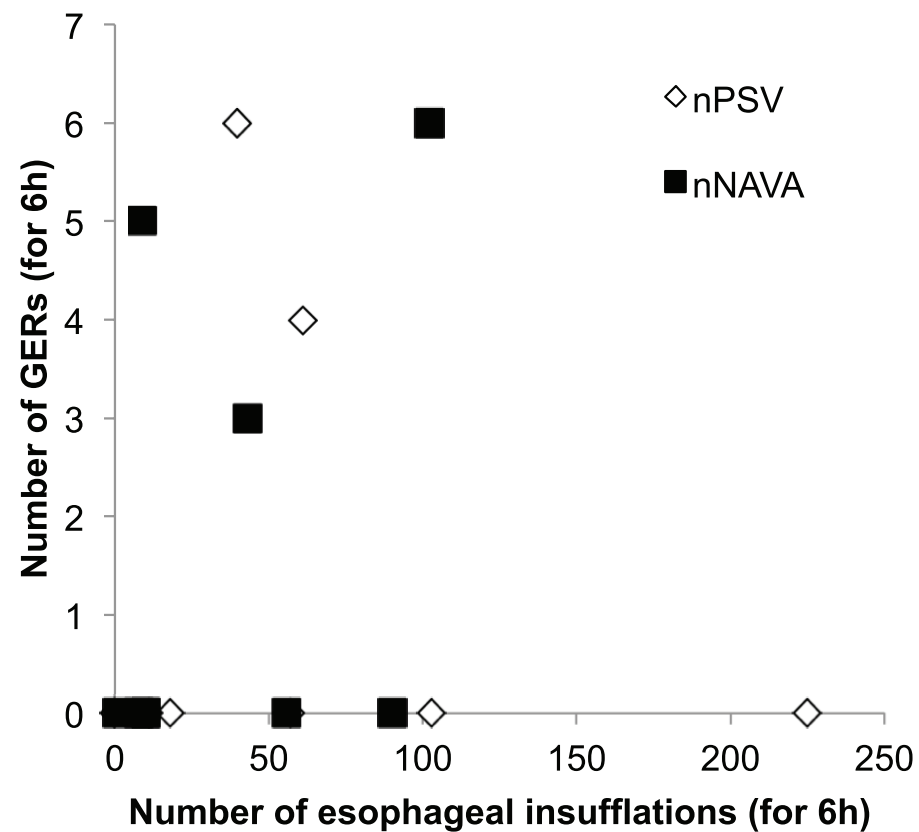**B**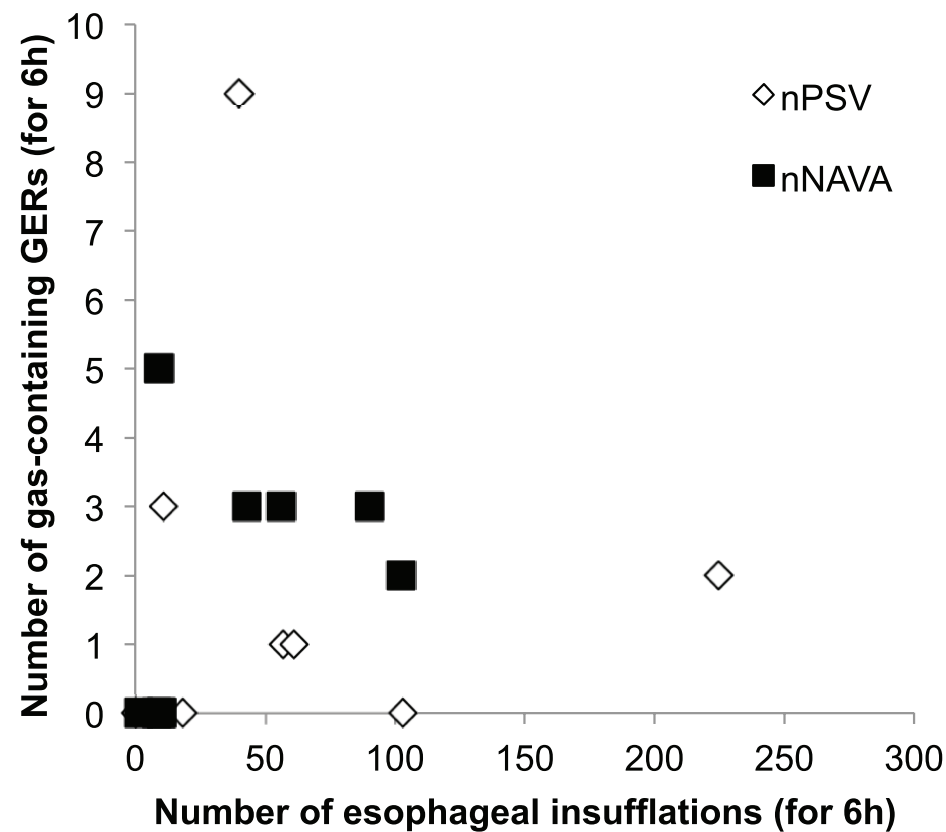

Supplement: S4 Fig — (PDF) [file pone.0146742.s004.pdf]
