# Supplementary material for: Inhibitory Effect of Nasal Intermittent Positive Pressure Ventilation on Gastroesophageal Reflux
Source: PLoS One. 2016 Jan 19;11(1):e0146742. doi: 10.1371/journal.pone.0146742 (PMC4718652; doi:10.1371/journal.pone.0146742)
Supplement: S1 Table — (DOCX) [file pone.0146742.s005.docx]

**S1 Table**

**Total number and physicochemical characteristics of gastro-esophageal refluxes (GERs) for each lamb during the six-hour ventilation period under control, nasal Pressure Support Ventilation and nasal Neurally-Adjusted Ventilatory Assist conditions.**

|  | Control | | | | | | nPSV | | | | | | nNAVA | | | | | |
| --- | --- | --- | --- | --- | --- | --- | --- | --- | --- | --- | --- | --- | --- | --- | --- | --- | --- | --- |
|  | Number  of GER*^c^* | pH | | Composition | | | Number  of GER*^c^* | pH | | Composition | | | Number  of GER*^c^* | pH | | Composition | | |
|  |  | WA  (%) | Alk  (%) | Liquid  (%) | Gaseous  (%) | Mixed  (%) |  | WA  (%) | Alk  (%) | Liquid  (%) | Gaseous  (%) | Mixed  (%) |  | WA  (%) | Alk (%) | Liquid  (%) | Gaseous  (%) | Mixed  (%) |
| Lamb 1 | 3 | 0 | 100 | 100 | 0 | 0 | 0 | 0 | 0 | 0 | 0 | 0 | 3 | 0 | 0 | 0 | 100 | 0 |
| Lamb 2 | 1 | 0 | 0 | 0 | 100 | 0 | 1 | 0 | 0 | 0 | 100 | 0 | 5 | 100 | 0 | 0 | 0 | 100 |
| Lamb 3 | 4 | 100 | 0 | 0 | 25 | 75 | 0 | 0 | 0 | 0 | 0 | 0 | 0 | 0 | 0 | 0 | 0 | 0 |
| Lamb 4 | 75 | 92 | 8 | 89 | 1 | 10 | 4 | 100 | 0 | 75 | 0 | 25 | 8 | 29 | 71 | 75 | 25 | 0 |
| Lamb 5 | 5 | 0 | 0 | 0 | 100 | 0 | 0 | 0 | 0 | 0 | 0 | 0 | 0 | 0 | 0 | 0 | 0 | 0 |
| Lamb 6 | 5 | n/a | n/a | 0 | 0 | 100 | 3 | n/a | n/a | 0 | 100 | 0 | 1 | n/a | n/a | 0 | 100 | 0 |
| Lamb 7 | 24 | 100 | 0 | 29 | 13 | 58 | 9 | 100 | 0 | 0 | 33 | 67 | 3 | 100 | 0 | 0 | 0 | 100 |
| Lamb 8 | 10 | 0 | 100 | 20 | 10 | 70 | 0 | 0 | 0 | 0 | 0 | 0 | 0 | 0 | 0 | 0 | 0 | 0 |
| Lamb 9 | 1 | 100 | 0 | 100 | 0 | 0 | 2 | 0 | 0 | 0 | 100 | 0 | 0 | 0 | 0 | 0 | 0 | 0 |

nPSV: nasal Pressure Support Ventilation; nNAVA: nasal Neurally-Adjusted Ventilatory Assist; GER: gastroesophageal reflux; WA: weakly acid; Alk: Alkaline; n/a: not available (= missing values due to technical difficulties).
