# Supplementary material for: Inhibitory Effect of Nasal Intermittent Positive Pressure Ventilation on Gastroesophageal Reflux
Source: PLoS One. 2016 Jan 19;11(1):e0146742. doi: 10.1371/journal.pone.0146742 (PMC4718652; doi:10.1371/journal.pone.0146742)
Supplement: S1 Text — (DOCX) [file pone.0146742.s006.docx]

**S1 Text**

**Data Analysis**

**Esophageal insufflations during nIPPV**

Frequency of esophageal insufflations. MII-pH recordings were also used to count the number of esophageal insufflations during one minute, every 10 minutes of the six-hour recording during no-nIPPV, nPSV and nNAVA. The esophageal insufflation index, defined as the number of esophageal insufflations per hour, as well as the percentage of respiratory cycles with esophageal insufflation, were calculated for the entire recording session in each respiratory condition. The effect of the state of alertness on esophageal insufflations was also assessed. The percentage of respiratory cycles with gas insufflation was first used to analyze the effect of wakefulness and QS. Then, given the paucity and shortness of AS epochs, a different analysis was performed to assess the effect of AS on esophageal insufflations. In every lamb with at least one epoch of AS, the percentage of respiratory cycles with gas insufflation was first counted on each epoch of AS. Each count was then normalized to one minute and a similar count was performed on the one-minute epoch of QS immediately preceding the given epoch of AS.

Effects of esophageal insufflations on GERs. A correlation analysis was performed to test whether the number of esophageal insufflations was associated with the total number of GERs or the number of gas-containing (gaseous + mixed) GERs observed during the six-hour recording with nPSV and nNAVA.

Effect of active laryngeal closure on esophageal insufflations. Lastly, the potential effect of inspiratory EAta, i.e., active glottal closure against ventilator insufflations, on diverting the insufflated gas into the esophagus during nPSV was assessed. Following confirmation that EAta was less frequent during AS, the % of respiratory cycles with esophageal insufflations was compared between QS and AS. Such an assessment could not be performed in nNAVA, due to the absence of inspiratory EAta as previously reported [1].

**References**

1. Hadj-Ahmed MA, Samson N, Bussières M, Beck J, Praud JP. Absence of inspiratory laryngeal constrictor muscle activity during nasal neurally adjusted ventilatory assist in newborn lambs. J Appl Physiol. 2012;113: 63-70.
