# Supplementary material for: Inhibitory Effect of Nasal Intermittent Positive Pressure Ventilation on Gastroesophageal Reflux
Source: PLoS One. 2016 Jan 19;11(1):e0146742. doi: 10.1371/journal.pone.0146742 (PMC4718652; doi:10.1371/journal.pone.0146742)

**S2 Text**

**Results**

**Esophageal insufflations during nIPPV**

Frequency of esophageal insufflations. No gas swallows were observed in control condition in any of the lambs. Conversely, esophageal insufflations were observed in both nPSV and nNAVA in most lambs, with no significant overall differences in the esophageal insufflation index between nPSV [40 (11, 61) h^-1^] and nNAVA [10 (9, 56) h^-1^] (p = 0.8) (Figure 3A). This corresponded to a low percentage of respiratory cycles with esophageal insufflation, which again was not significantly different between nPSV [5 (2, 8)%] and nNAVA [1 (1, 8)%] (p = 0.9), even when QS and QW were taken into account (Figure A – panel A below). Moreover, in both nPSV and nNAVA, the percentage of respiratory cycles with esophageal insufflation was higher in AS [30 (11, 50)% (n = 5) and 39 (15, 68)% (n = 4), respectively] comparatively to QS [9 (9, 11)% and 1 (0, 3)%, respectively] (p = 0.07 in both cases) (see Figure A – panel B below).

Effects of esophageal insufflations on GERs. Despite the insufflation of air into the esophagus during nPSV and nNAVA, no increase was observed in nPSV or nNAVA with regard to the number of gas-containing (gaseous + mixed) GERs compared to the control condition (p = 0.1) (Figure 4). In addition, no significant association was found between the number of total GERs, or the number of gas-containing GERs, and the number of esophageal insufflations (see S7, which illustrates the relationship between the number of esophageal insufflations and the number of GERs and gas-containing GERs in each lamb).

Furthermore, esophageal insufflations did not increase abdominal circumference during nIPPV [- 0.1 (- 0.8, 0) cm in nPSV and + 0.3 (- 0.2, + 1.1) cm in nNAVA *vs.* - 0.8 (- 1.4, - 0.5) cm in control condition, p = 0.1], suggesting the absence of gastric distension during nIPPV (measurements performed in the last six lambs only).

Effect of active laryngeal closure on esophageal insufflations. Only four lambs presented at least one AS epoch of 60 seconds with analyzable EAta, hence with a total time of 23.5 minutes for AS (and hence of QS) studied. The most frequent pattern of esophageal insufflations and EAta recordings obtained during two successive periods of QS and AS is illustrated in Figure 5. We first confirmed that the percentage of respiratory cycles with inspiratory EAta (an evidence of active laryngeal closure) in nPSV was lower in AS compared to QS [54 (48, 61)% vs. 100 (98, 100)% respectively, p = 0.07; n = 4] (Figure 6A). This enabled to assess the effect of laryngeal closure on esophageal insufflations during nPSV. Conversely to results on inspiratory laryngeal closure, the % of respiratory cycles with esophageal insufflations was higher in AS [30 (11, 50) % compared to QS [4 (1, 15) % in QS, p = 0.07; n = 4] (Figure 6B), suggesting that esophageal insufflations are not a result of active laryngeal closure.

**Figure A – Percentage of respiratory cycles with esophageal insufflation in nPSV vs. nNAVA, according to the state of alertness**


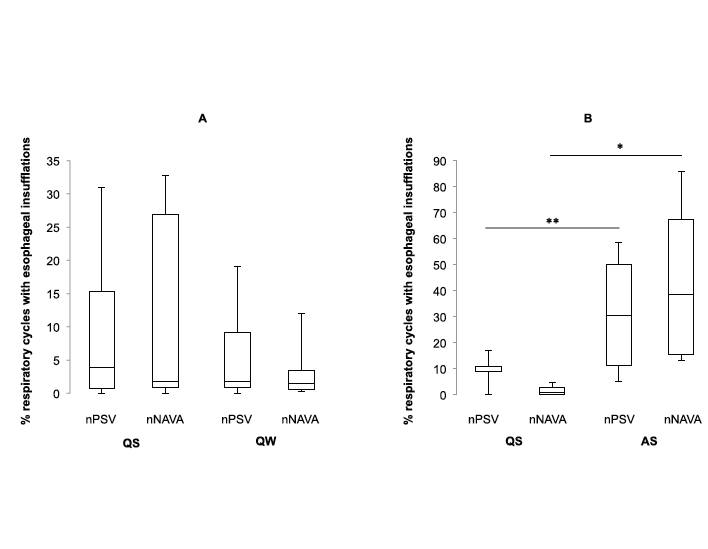

Supplement: S2 Text — (DOCX) [file pone.0146742.s007.docx]
